# Plastic/Natural Fiber Composite Based on Recycled Expanded Polystyrene Foam Waste

**DOI:** 10.3390/polym14112241

**Published:** 2022-05-31

**Authors:** Wilasinee Sriprom, Adilah Sirivallop, Aree Choodum, Wadcharawadee Limsakul, Worawit Wongniramaikul

**Affiliations:** Integrated Science and Technology Research Center, Faculty of Technology and Environment, Phuket Campus, Prince of Songkla University, Kathu, Phuket 83120, Thailand; adey.siriwallop@gmail.com (A.S.); aree.c@phuket.psu.ac.th (A.C.); wadcharawadee.n@phuket.psu.ac.th (W.L.); worawit.won@phuket.psu.ac.th (W.W.)

**Keywords:** natural fiber, recycled expanded polystyrene foam, natural fiber-recycled plastic composites, mechanical properties, dissolution

## Abstract

A novel reinforced recycled expanded polystyrene (r-EPS) foam/natural fiber composite was successfully developed. EPS was recycled by means of the dissolution method using an accessible commercial mixed organic solvent, while natural fibers, i.e., coconut husk fiber (coir) and banana stem fiber (BSF) were used as reinforcement materials. The treatment of natural fibers with 5% (*w*/*v*) sodium hydroxide solution reduces the number of –OH groups and non-cellulose components in the fibers, more so with longer treatments. The natural fibers treated for 6 h showed rough surfaces that provided good adhesion and interlocking with the polymer matrix for mechanical reinforcement. The tensile strength and impact strength of r-EPS foam composites with treated fibers were higher than for non-filled r-EPS foam, whereas their flexural strengths were lower. Thus, this study has demonstrated an alternative way to produce recycled polymer/natural fiber composites via the dissolution method, with promising enhanced mechanical properties.

## 1. Introduction

Expanded polystyrene (EPS) foam is a thermoplastic that has been used to make a wide variety of consumer products. Due to its low thermal conductivity, high compressive strength, extremely light weight, versatility, durability, and moisture resistance, it is often used as an insulator, in lightweight protective packaging, and in food packaging. With the increased use of EPS foam, its waste has also increasingly accumulated around the world. However, discarded foam waste is non-biodegradable and resistant to photolysis [1]. It can break down when exposed to sunlight, rain, and ocean water (especially in tropical waters) into its constituents, including styrene monomers [2] that are classified as possible human carcinogens [3]. Styrene trimers may also increase thyroid hormone levels [4].

Fortunately, EPS foam waste has been reported as an excellent material for recycling [5]. To recycle polystyrene (PS) foams, they are typically cleaned first and then densified to shippable logs using a thermal treatment [6]. The densified or compressed PS obtained is then simply chopped up, heated, and recast into plastic pellets that can be used as raw materials for plastic products [7]. Dissolution of PS foam with a suitable solvent has become an alternative method for waste volume reduction or recycling, as it is one of the least costly alternatives and uses less energy than melting or compressing the waste [8]. Various organic solvents, such as toluene, acetone, limonene, and other liquid hydrocarbons have been reported for PS foam dissolution [5,8,9,10,11]. The dissolved EPS can be used for packaging, similar to the brand new polymer [12], and various products can be produced, e.g., nanofibers [13,14], or polymer–cement composites [5].

The loss of mechanical properties of the polymer during recycling has been reported, and reinforcement with glass fibers or natural fibers is applied to conquer this problem and extend the potential applications of recycled plastic materials [15,16,17]. There are recent studies on the characterization of wood-recycled plastic composites, showing potential as alternative materials [18,19]. Several kinds of natural fibers [20] have been reported as reinforcing fillers, e.g., banana/jute/flax fiber [21,22], kenaf/coir [23,24,25], and bagasse/Napier grass fiber–polyester composites [26,27]. Nevertheless, most of the previous studies have reinforce virgin plastic (polymer pellets) instead of recycled plastic, which may influence mechanical properties of the filled composite.

The aim of this work was to investigate the feasibility to produce a novel composite using EPS recycled via dissolution (r-EPS) and reinforced with natural fiber. A commercial grade thinner containing mixed organic solvents was used, along with acetone, as a solvent to recycle the EPS. Coconut husk fiber (coir) and banana stem fiber (BSF) were incorporated into r-EPS to enhance the mechanical properties of the composite. The coir and BSF were first treated with an alkaline solution and mixed with r-EPS to various filler loadings in the composite sheets. The mechanical properties of both r-EPS and the composite sheets were then evaluated.

## 2. Materials and Methods

### 2.1. Materials

The used and clean expanded polystyrene (EPS) foam boards were collected from Prince of Songkla University, Phuket Campus. It was analyzed using gel permeation chromatography (GPC) (Shodex Standard SM-105) equipped with Shodex GPC KF-806 M and KF-803 L (300 mm Length × 8.0 mm ID) using RI-Detector, obtaining the number average molecular weight ($\bar{M_{n}}$) (120,491 g/mol) and the polydispersity index (PDI) (2.17). THF was used as the eluent at a flow rate of 1.0 mL/min at 40 °C. The GPC system was calibrated with polystyrene (PS) standards, with molecular weights ranging from 3790 to 3,053,000 g/mol. Acetone (99.98%) was purchased at the highest purity available from Fisher Chemical (Loughborough, England). Commercial thinner containing mixed organic solvents, including toluene (70%), acetone (15.4%), ethyl acetate (4.9%), 2-butoxyethanol (3.9%), 2-propanol (2.9%), and 2-methyl-1-propanol (2.9%), was supplied by TOA Paint (Thailand) Co., Ltd. (Samutprakan, Thailand) [28]. Coconut and banana fibers were prepared from coconut husks and banana stems collected from a fruit garden in Phuket, Thailand.

### 2.2. Dissolution of EPS Foam

An EPS foam board was broken into small pieces (30 g) before dissolving in 200 mL of the mixed organic solvents (thinner: acetone in a 3:1 volume ratio). The mixture was constantly stirred at 750 rpm for 4 h at room temperature to obtain a homogenous solution. The dissolved EPS solution (r-EPS) was used for the preparation of natural fiber-reinforced recycled EPS foam composites.

### 2.3. Preparation and Characterization of Natural Fiber

Coconut husk fiber (coir) and banana stem fiber (BSF) were cleaned by washing with tap water and then dried in an oven at 100 °C for 24 h. They were chopped into small pieces and sieved to a length of 1 to 3 mm. An alkali treatment was applied on both coir and BSF separately by immersing the chopped fibers in 5% aqueous sodium hydroxide (NaOH) solution for 6, 12, or 24 h at room temperature. The treated fibers were vacuum filtered and washed with distilled water until the water became neutral (pH = 7). Then, the treated fibers were dried in an oven at 80 °C for 24 h.

The chemical functionality of treated and untreated natural fibers was evaluated by Fourier transform infrared spectroscopy (FTIR, Perkin-Elmer Frontier). Transmittance was measured over a range from 4000 to 600 cm^−1^. The surface topography and compositions of treated and untreated natural fibers were observed by using a scanning electron microscope, SEM Quanta 400, operated at 20 kV at 1000×.

### 2.4. Preparation of Natural Fiber-Reinforced Recycled EPS Foam Composites

Both untreated and treated coir and BSF were mixed with r-EPS at 2%, 5%, and 10% by total weight to obtain natural fiber-reinforced recycled EPS foam composites. Next, the composites were slowly poured to fill a 25 × 150 mm Petri dishes and then set aside to dry at room temperature in the fume hood. The mass of samples was measured and recorded until the constant mass was obtained within 72 h. With the ease of preparing the composites using these conditions, the solvent blend could be recovered for further use as a solvent for EPS recycling.

### 2.5. Mechanical Properties of Natural Fiber-Reinforced Recycled EPS Foam Composites

The tensile strength, flexural strength, and impact strength of recycled EPS foam and recycled EPS foam natural fiber composites were investigated, as summarized in Table 1. Each sample was cut into three specimens, and the same test was performed on these replicates. The average test results with standard deviations (SD) are reported.

Tensile testing: Each sample was cut into three dumbbell-shaped specimens (gauge length, L_0_ of 60 mm). The tensile test was conducted using the Instron (Model 5566) universal testing machine with a 1kN load cell at 1 mm/min crosshead speed. The test was continued until tensile failure occurred.

Flexural testing: Three-point bending flexural testing was carried out with an Instron universal testing machine (Model 55R4502). The load cell and the crosshead speed were 1 kN and 1.13 mm/min, respectively, while the support span was 42 mm. The rectangular specimens had 80mm (L) × 12.7 mm (W) × 2.3 mm (T) dimensions.

Impact testing: Notched Izod impact testing was performed according to the ASTM D256 standard method for determining the impact resistance of plastic samples. The testing specimen was 65 mm (L) × 13 mm (W) × 2.5 mm (T). The depth under the notch of the specimen was 10 mm. A pendulum energy of 1 joule was employed in the testing.

## 3. Results and Discussion

### 3.1. Characterization of Natural Fibers

**Figure 1:**
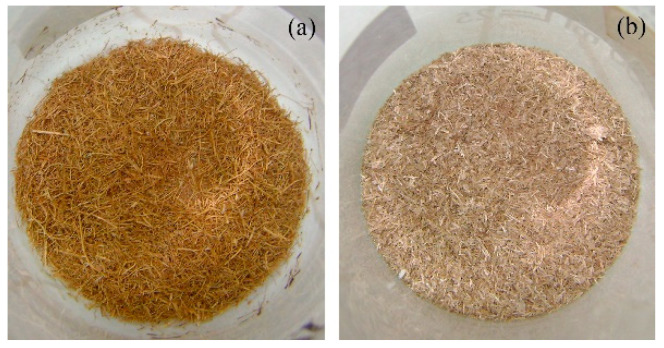
Treated fibers. (**a**) coir, and (**b**) BSF.

**Figure 2:**
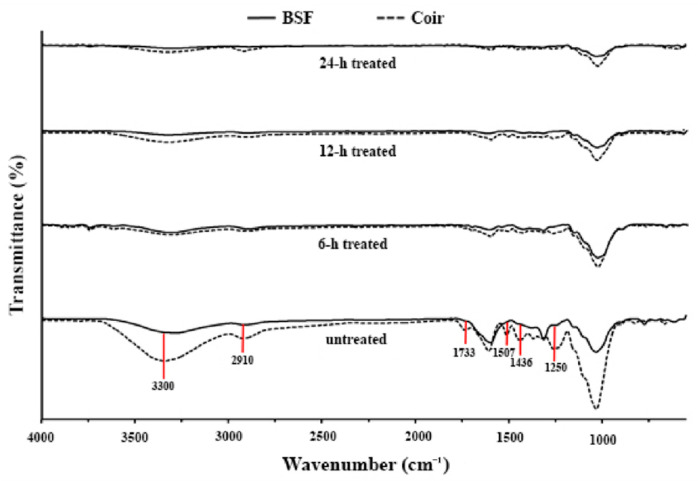
FTIR spectra of untreated and alkali-treated BSF and coir.

The treated natural fibers are shown in Figure 1, and their chemical functionalities are shown in Figure 2. The FTIR of both untreated BSF and coir showed characteristic peaks of cellulose, hemicellulose, and lignin. The absorption bands at 3300 and 2910 cm^−1^ indicate hydroxyl groups (–OH) and –CH stretching vibrations, respectively, from the chemical structures of cellulose and hemicellulose. The peak at 1733 cm^−1^ was attributed to C=O stretching vibrations of the acetyl group in the hemicelluloses. Adsorption at 1507, 1436, and 1250 cm^−1^ was attributed to C=C aromatic symmetrical stretching, HCH and OCH in plane bending vibrations, and C-O stretching vibrations of the acetyl groups, respectively, and these are typical absorption peaks of lignin [29]. After alkali treatment of BSF and coir, all absorption peaks decreased with treatment time. The absorption peaks around 1733–1250 cm^−1^ of both fibers disappeared after 24 h of treatment, indicating that lignin and hemicellulose might be removed, matching some previous reports [20,30,31,32].

**Figure 3:**
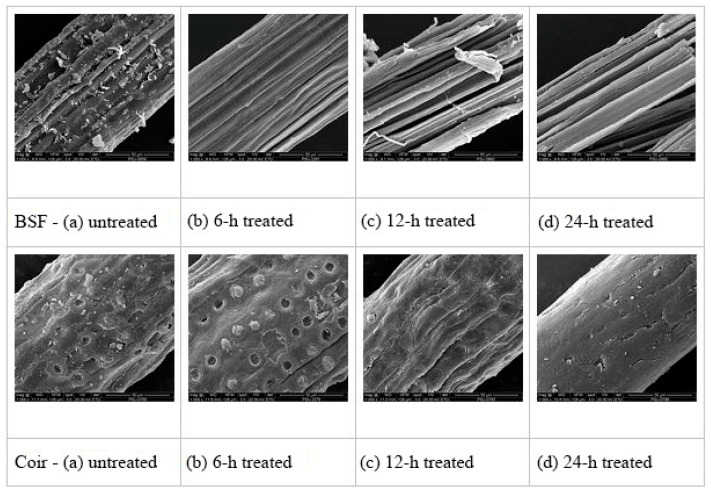
SEM micrographs (at 1000×) of BSF and coir: (**a**) untreated, (**b**) 6 h treated, (**c**) 12 h treated, and (**d**) 24 h treated.

The surface topography of BSF and coir, both untreated and treated with 5% NaOH solution, was investigated, and the SEM micrographs are shown in Figure 3. The surfaces of untreated BSF and coir were not smooth, spread with nodes, and covered with irregular strips (Figure 3a) that may represent lignin, hemicellulose, or impurities [33]. After alkali treatment of both fibers for 6 h, the layer of substances on the fiber surface seemed to be removed (Figure 3b), matching the FTIR results indicating that lignin and hemicellulose contents were decreased. Some holes and rough surfaces were obviously observed, especially for coir fiber, that could improve the mechanical interlocking of the fiber and polymer matrix [33,34,35]. However, after alkali treatments for 12 or 24 h, the surfaces of the fibers had become smoother (Figure 3c,d), so that potentially, the interfacial bonding with the polymer matrix would be weaker than with the 6 h treatment of the fibers. It can be expected that the alkali treatment of fibers for longer than 6 h may cause damage by removing hemicelluloses, lignin, and bound cellulose from the fibers, which weakens the fiber strength [32]. Therefore, 6 h treated fiber was considered the most suitable for producing fiber-reinforced polymer composites, possibly having a good interfacial bonding with the polymer matrix and a desirable amount of cellulose exposed on the fiber surfaces.

The mechanical and physical properties of coir and banana fiber have been reported in the literature, as summarized in Table 2 [36,37,38,39]. However, Yue et al. [40] reported that the mechanical properties of many natural plant fibers may vary, to a large extent due to inappropriate measurement.

### 3.2. Characterization of Recycled EPS Foam/Natural Fiber Composites

Composite materials of r-EPS foam with BSF and coir at 2%, 5%, and 10% by weight were prepared using both the untreated (u) and the treated (t) fibers (treatment with 5% NaOH aqueous solution for 6 h). Composite sheets with 3 mm thickness and 130 mm diameter were obtained. Their weights were increased from the total mass of EPS and fiber by ~6%, which might be attributed to organic solvent trapping during the curing process. The distribution of fibers in the EPS matrix and the mechanical properties tensile strength, flexural strength, and impact strength of the composites were then investigated.

#### 3.2.1. The Distribution of Fibers in r-EPS

**Figure 4:**
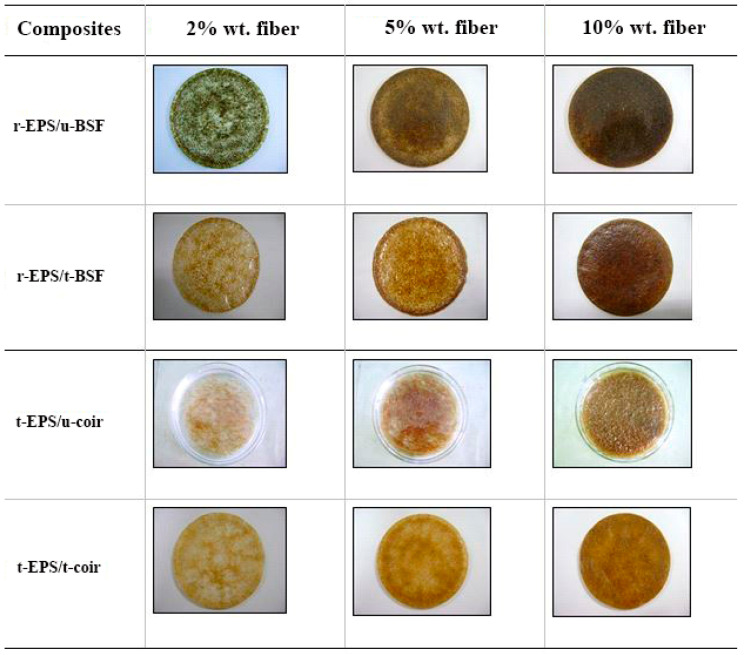
Digital photographs showing the distribution characteristics of fibers in the recycled EPS (r-EPS) foam/natural fiber composite sheets prepared at different fiber loadings.

Images of both treated and untreated BSF and coir in r-EPS at different fiber loadings are shown in Figure 4. The untreated fibers were gathered mostly at the center of the composite sheet, whereas the treated fibers were distributed more evenly in the matrix. This is because of the improved fiber-polymer matrix adhesion [33,34,35]. At the same mass of the fibers mixed in the composite, the BSF spreads more thoroughly within the composite sheet than does the coir, due to it being a greater density fiber (BSF has the smaller volume) [41]. Increasing the fiber loading caused the fibers to be more evenly distributed all over the composite sheet, and the fibers were aligned in the polymer matrix. However, it was found that the composite of EPS with 10% *w*/*w* untreated coir had some excess fibers appearing on the polymer surfaces. The results confirmed that the wettability of fibers by polymer solution was enhanced by the alkali treatment, as the –OH groups were modified to –O^−^Na^+^ groups [32,42], resulting in the reduction in the polarity of the fibers [43]. Consequently, the treated fibers more readily form stronger interfacial adhesion with the polymer matrix [36,43,44], so they dispersed throughout the composite sheet better than the untreated fibers. Maximizing the interfacial adhesion between the fibers and the polymer matrix would provide the final composite material with the highest strength [34,45,46,47,48].

#### 3.2.2. Mechanical Properties

**Figure 5:**
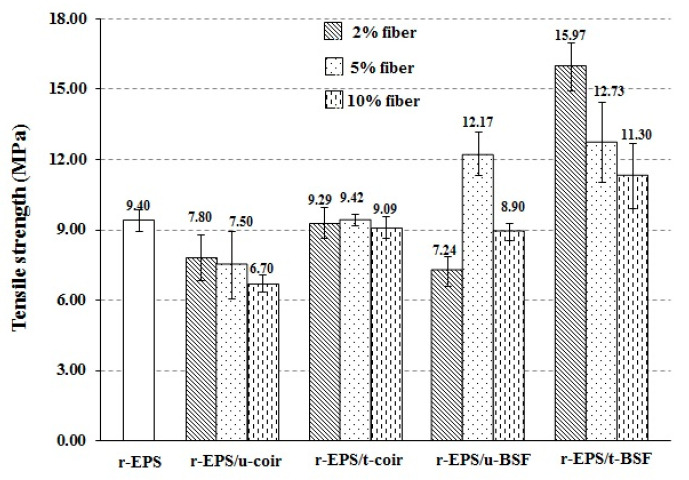
Tensile strengths of r-EPS composites with untreated and treated BSF or coir fibers at 2%, 5%, or 10% wt. loadings.

The tensile strength, flexural strength, and impact strength of r-EPS/natural fiber composites and r-EPS (without fiber) were evaluated and compared. The composites with untreated coir (r-EPS/u-coir) had lower tensile strength than the r-EPS, whereas composites with treated coir (r-EPS/t-coir) provided similar tensile strength. On the other hand, the tensile strength of all composites with treated BSF (r-EPS/t-BSF) was higher than that of r-EPS, whereas the composites with untreated BSF (r-EPS/u-BSF) showed reduced tensile strength (Figure 5). As described in the previous section, the alkali treatment of fiber not only caused the fiber surfaces to be less polar, but also increased surface roughness, enabling a stronger interlocking of fibers with the polymer matrix. Therefore, the alkali treated fibers should provide better interfacial adhesion with the polymer matrix than untreated fibers [27,34,36,44,48]. On the other hand, the addition of untreated fibers resulted in lower tensile strength, due to the unevenness of fiber distribution in the matrix and the weak adhesion of fibers. Furthermore, it was found that the higher content of treated BSF distributed in the polymer matrix decreased the tensile strength of the composites because of fiber agglomeration by fiber–fiber interactions, more so at higher fiber loadings. Similar results were observed by Ibrahim et al. [45] and Ramesh et al. [49].

**Figure 6:**
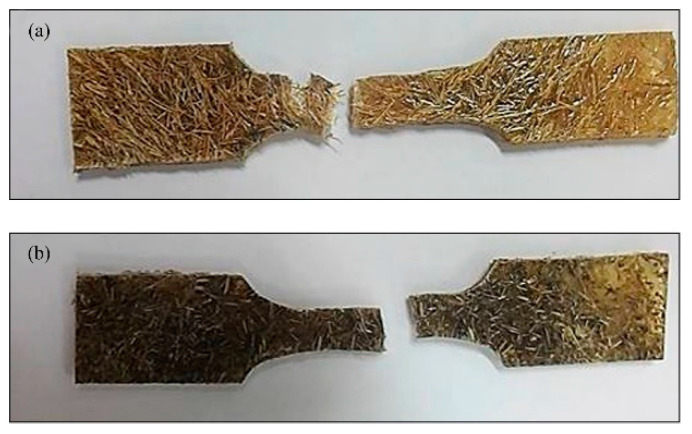
Specimens after tensile testing: (**a**) r-EPS/5% t-coir, and (**b**) r-EPS/5% t-BSF.

In addition, it can be observed that the tensile strength of the treated BSF composite (r-EPS/t-BSF) was higher than that of the coir composite (r-EPS/t-coir). This indicates that the BSF provides better reinforcement in the composite than coir, potentially because the BSF contains more crystalline cellulose [20]. The BSF fibers are also less thick, which provides better wettability (fewer gaps at the interface) between the fibers and the polymer matrix. This was confirmed by the tear surfaces of dumbbell-shaped specimens after tensile testing of both r-EPS/5% t-coir and r-EPS/5% t-BSF (Figure 6). The composite of treated coir showed fibers slipping out from the polymer matrix more frequently than what was observed in the composite filled with treated BSF.

**Figure 7:**
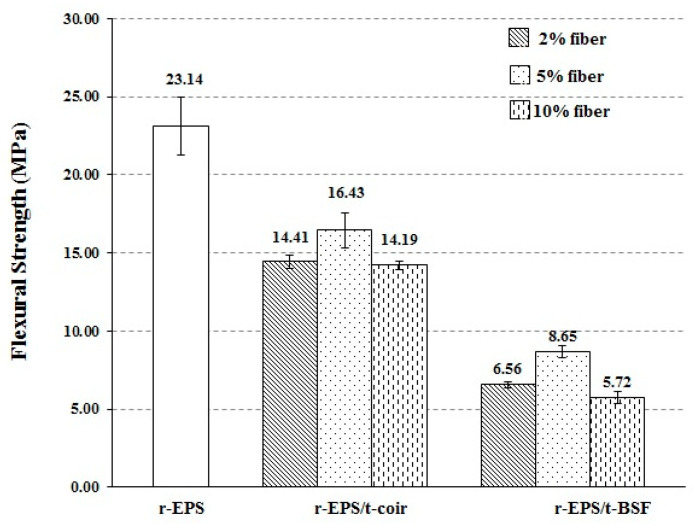
Flexural strength of r-EPS composites with treated BSF or coir at 2%, 5%, or 10% wt. fiber loadings.

The flexural strength of treated BSF and coir reinforced r-EPS composites with different fiber loadings was evaluated by three-point bending flexural testing. The flexural strength of both the r-EPS/t-coir and r-EPS/t-BSF composites was smaller than that of the r-EPS (Figure 7), indicating that the natural fibers diminished bending resistance. This is possibly because the orientation of fibers may be at right angles to the direction of the force acting on them [49,50]. When compared with the tensile test discussed previously, the treated fibers added in the r-EPS enhanced the tensile strength of the material, as the pulling force is at right angles to the bending force, confirming the parallel arrangement of the fibers to the pulling direction, or the perpendicular arrangement to the bending direction. In addition, the flexural strength of r-EPS/t-coir was found to be greater than that of r-EPS/t-BSF at the same fiber loadings. This may be attributed to the fact that most of the treated coir in the composite was aligned in the direction of the bending force [47,51], more so than in the treated BSF. The flexural strength of the composites also increased as the fiber content increased from 2% to 5%, but it decreased to the lowest level for 10% of either treated fiber. This is likely due to the less uniform fiber distribution with greater fiber loading in the polymer matrix. Similar results were obtained for the tensile test, as described previously.

**Figure 8:**
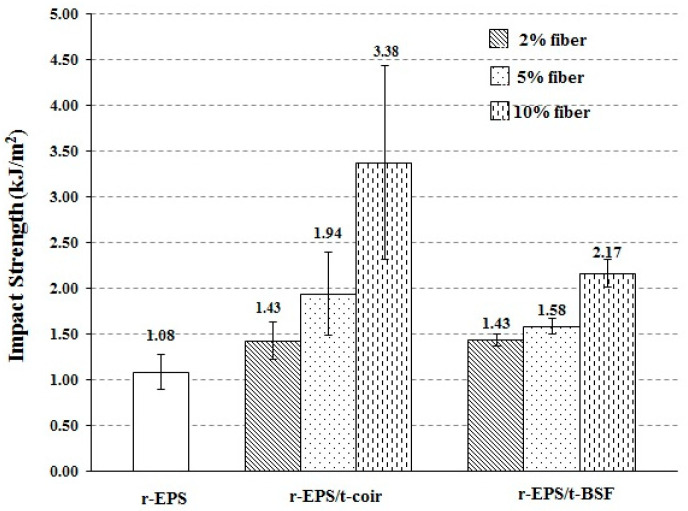
Impact strength of r-EPS composites with treated BSF or coir at 2%, 5%, or 10% wt. fiber loadings.

The impact strength of the composites was found to be increased from that of r-EPS (Figure 8), and it increased with the loading of treated fibers. Moreover, the r-EPS/t-coir composite showed higher impact strength than the r-EPS/t-BSF composite at the same loading. This indicates that the coir may absorb greater force during the impact test than the BSF. Since the coir is larger in size or lower in density than the BSF, the greater volume of the coir presents in the composite at the same mass added [41].

## 4. Conclusions

This study has demonstrated the production of r-EPS foam composites reinforced with coir and BSF by the dissolution process. The natural fibers were treated with 5% (*w*/*v*) NaOH for 6, 12,, or 24 h. The FTIR analyses revealed that the number of hydroxyl groups and non-cellulose components in the fibers decreased with treatment time, and SEM imaging showed that alkali treatment modified the fiber structure. The 6 h treated BSF (2% wt.) reinforced r-EPS foam composite provided the greatest increase in tensile strength by about 70%, whereas r-EPS foam composite reinforced with 10% wt. of 6 h treated coir showed the maximum increase in impact strength, by about 210% compared to r-EPS. This was due to the good adhesion and interlocking of treated fibers with the polymer matrix. This study presented an alternative method to produce recycled polymer/natural fiber composites via the dissolution method, with promising enhanced mechanical properties.

## Figures and Tables

**Table 1:** Materials prepared from recycled EPS to evaluate the mechanical properties.

Material *	Tensile Strength	Flexural Strength	Impact Strength
r-EPS	✓	✓	✓
r-EPS/u-coir (2, 5, and 10%)	✓	-	-
r-EPS/u-BSF (2, 5, and 10%)	✓	-	-
r-EPS/t-coir (2, 5, and 10%)	✓	✓	✓
r-EPS/t-BSF (2, 5, and 10%)	✓	✓	✓

* r-EPS = recycled expanded polystyrene foam; u-coir = untreated coir; t-coir = treated coir; u-BSF = untreated banana stem fiber; t-BSF = treated banana stem fiber.

**Table 2:** Mechanical and physical properties of the fibers.

Properties	Coir	Banana
Diameter (μm)	150–250 [36]	100–250 [36]
Density (g/cm^3^)	1.2 [36,37]	0.8 [36]
Tensile Strength (MPa)	175 [36], 131–220 [37]	161.8 [36]
Young’s modulus (GPa)	4–6 [36,37]	8.5 [36]
Elongation at break (%)	30 [36], 15–30 [37]	2.0 [36]
Surface energy (mJ/m^2^)	35.1 ± 1.3 [38]	39.49 [39]

## Data Availability

All data are available from the corresponding author on reasonable request.
